# A FRET-Based Real-Time PCR Assay to Identify the Main Causal Agents of New World Tegumentary Leishmaniasis

**DOI:** 10.1371/journal.pntd.0001956

**Published:** 2013-01-03

**Authors:** Pablo Tsukayama, Jorge H. Núñez, Maxy De Los Santos, Valeria Soberón, Carmen M. Lucas, Greg Matlashewski, Alejandro Llanos-Cuentas, Marianela Ore, G. Christian Baldeviano, Kimberly A. Edgel, Andres G. Lescano, Paul C. F. Graf, David J. Bacon

**Affiliations:** 1 Department of Parasitology, U.S. Naval Medical Research Unit No. 6 (NAMRU-6), Lima, Peru; 2 Department of Microbiology and Immunology, McGill University, Quebec, Canada; 3 Leishmaniasis Working Group, Instituto de Medicina Tropical ‘Alexander von Humboldt’, Universidad Peruana Cayetano Heredia, Lima, Peru; 4 Hospital Militar Central, Lima, Perú; 5 Microbiology, Immunology, and Molecular Diagnostics Laboratory Department, Naval Medical Center San Diego, San Diego, California, United States of America; 6 Naval Research Laboratory, Washington, D.C., United States of America; Hebrew University-Hadassah Medical School, Israel

## Abstract

In South America, various species of *Leishmania* are endemic and cause New World tegumentary leishmaniasis (NWTL). The correct identification of these species is critical for adequate clinical management and surveillance activities. We developed a real-time polymerase chain reaction (PCR) assay and evaluated its diagnostic performance using 64 archived parasite isolates and 192 prospectively identified samples collected from individuals with suspected leishmaniasis enrolled at two reference clinics in Lima, Peru. The real-time PCR assay was able to detect a single parasite and provided unambiguous melting peaks for five *Leishmania* species of the *Viannia* subgenus that are highly prevalent in South America: *L.* (*V.*) *braziliensis, L.* (*V.*) *panamensis, L.* (*V.*) *guyanensis, L.* (*V.*) *peruviana* and *L.* (*V.*) *lainsoni*. Using kinetoplastid DNA-based PCR as a gold standard, the real-time PCR had sensitivity and specificity values of 92% and 77%, respectively, which were significantly higher than those of conventional tests such as microscopy, culture and the leishmanin skin test (LST). In addition, the real-time PCR identified 147 different clinical samples at the species level, providing an overall agreement of 100% when compared to multilocus sequence typing (MLST) data performed on a subset of these samples. Furthermore, the real-time PCR was three times faster and five times less expensive when compared to PCR - MLST for species identification from clinical specimens. In summary, this new assay represents a cost-effective and reliable alternative for the identification of the main species causing NWTL in South America.

## Author Summary

Leishmaniasis is a neglected disease with more than two million new human infections annually worldwide. Tegumentary leishmaniasis, cutaneous and mucocutaneous, is mainly caused by five *Leishmania* species of the *Viannia* complex in South America. Different species can cause disease with similar symptoms but have dissimilar prognoses and may need different therapeutic regimens. Identification of *Leishmania* species traditionally relies on the multilocus enzyme electrophoresis (MLEE) assay, but it can only be applied to culture-positive samples and takes at least six weeks of intense laboratory work. A reliable and rapid assay for species identification can be a valuable tool. Molecular assays are the fastest and most accurate way to identify the etiological agents causing leishmaniasis. This paper describes a novel real-time PCR assay for identification of the five main species that cause tegumentary leishmaniasis in the New World. The assay correctly identified each of these five species of *Leishmania* directly from clinical samples. Because of its reliability, speed and simplicity, this assay could be used for species identification in routine laboratory diagnosis of leishmaniasis in endemic regions.

## Introduction

The leishmaniases are a globally widespread group of vector-borne diseases that are endemic to 88 countries and affect an estimated 12 million people, with approximately 350 million people at risk worldwide [1]. Depending on the parasite species and host genetic background, infection can range from self-healing cutaneous ulcers to disfiguring mucocutaneous forms and lethal visceral disease [2]. In South America, New World tegumentary leishmaniasis (NWTL) is mainly caused by species of the *Viannia* complex. The most prevalent species are *L.* (*V.*) *braziliensis*, *L.* (*V.*) *panamensis*, *L.* (*V.*) *peruviana*, *L.*(*V.*) *guyanensis* and *L.* (*V.*) *lainsoni* [1]–[2]. In Peru, 70% of the country's region is affected with leishmaniasis, 96% of which are caused by three species: *L.* (*V.*) *braziliensis*, *L.* (*V.*) *peruviana* and *L.* (*V.*) *guyanensis* [2]–[3].

Diagnosis of leishmaniasis is based on criteria that consider epidemiological data, clinical features and laboratory test results [2]. Most laboratory methods are based on findings of the etiological agent microscopically or by culture; however, they have relatively low sensitivity and they do not identify the infecting species [2]. Serological tests and the leishmanin skin test (LST) have also been used as diagnostic tools but they do not distinguish between present and past infections, and their specificity is low in endemic areas [2]. A variety of molecular approaches have been developed. For instance, PCR methods based on the detection of kinetoplastid DNA (kDNA PCR) are highly sensitive due to the presence of thousands of copies of these sequences in the parasite. Thus, correct diagnosis of leishmaniasis should be based on clinical suspicion of leishmaniasis with a confirmed laboratory diagnosis, where the physician must take into account the reliability of the given laboratory result.

The correct diagnosis and characterization of the infecting parasite may be important for directing appropriate treatment and evolution of the disease [2]. For instance, patients infected with *L.* (*V.*) *braziliensis* have a higher risk of developing the disfiguring mucosal manifestations [4]. In addition, different species show varying response rates to therapeutic drugs [5]. Therefore, early identification of the etiological species may lead to improved patient management.

The identification of *Leishmania* species has been traditionally performed by multilocus enzyme electrophoresis (MLEE), for which mannose phosphate isomerase (*MPI*) is the only reliable marker for discrimination between the closely related species *L.* (*V.*) *braziliensis* and *L.* (*V.*) *peruviana* [6]. However, this technique presents several drawbacks: a) it can only be applied to culture-positive cases; b) it requires the isolation and mass growth of the parasite; and c) it is time-consuming, taking up to six weeks for definitive parasite identification. These limitations underscore the pressing need for improved identification methods that are fast, cost-effective and more reliable for the diagnosis and characterization of leishmaniasis cases [7].

Several real-time PCR and melting curve analysis using SYBR Green or fluorescence resonance energy transfer (FRET) probes in combination with kDNA or 18S rDNA amplification have recently been reported for the detection and identification of Old and New World *Leishmania* species. *L.* (*V.*) *braziliensis* has been the only New World *Leishmania* species primarily included in these assays [8]–[13]. Another study revealed that a SYBR Green-based real-time PCR assay targeting the conserved region of kDNA mini-circles was able to differentiate between *L.* (*Leishmania*) and *L.* (*Viannia*) at the complex level [14], although this assay did not distinguish species within each complex. Other molecular approaches such as PCR followed by restriction fragment length polymorphism (PCR-RFLP) [15]–[16], multilocus sequence typing (MLST) [17]–[18] or multiplex PCR [19] have been used to discriminate species within the *Viannia* complex. However, these approaches present a number of limitations, including laborious procedures, complex data interpretation and long processing times for species identification [8]–[10], [13]–[14], [17], [19].

We recently identified new species-specific genetic polymorphisms in the genes that confer the phenotypic variations in the MLEE assay [18]. A combination of sequencing of the *MPI* and 6-phosphogluconate dehydrogenase (*6PGD*) genes was sufficient to differentiate among seven closely related species causing New

World leishmaniasis [18]. In this study, we took advantage of these polymorphisms in order to devise a new real-time PCR assay based on FRET technology and melting curve analysis. The assay was highly sensitive and correctly identified each of the five species of *Leishmania* being evaluated. Because of its reliability, short turnaround time and simplicity, this assay could be used for species identification in routine laboratory diagnosis of leishmaniasis in endemic regions, thus allowing better management of patients affected with NWTL.

## Materials and Methods

### Source of Specimens and Ethics Statement

*Leishmania* reference strains were obtained from the World Health Organization (WHO) by Lucas *et al.* [3]. *Leishmania* isolates were previously obtained from patients enrolled using a written informed consent [3] and anonymized for this study. Isolates from the Instituto de Medicina Tropical “Alexander von Humboldt” (IMTAvH) samples were obtained from clinical cases seen in 2008 and sent to the U.S. Naval Medical Research Unit No. 6 (NAMRU-6) for diagnosis confirmation and species identification. Samples from the Hospital Militar Central (HMC) were collected by the Peruvian Army during an investigation of an outbreak (February-April 2010), and sent to NAMRU-6 for diagnosis confirmation and species identification. The analysis of both sets of samples was approved by the Institutional Review Board (IRB) of NAMRU-6 in compliance with all applicable federal regulations governing the protection of human subjects. All samples were anonymized before being sent to NAMRU-6.

### *Leishmania* Reference Strains and Isolates

Five *Leishmania* reference strains from the WHO and 59 well-characterized *Leishmania* strains were assessed to determine the melting curves for each *MPI* and *6PGD* locus. Strains and isolates belonged to species of the subgenus *Viannia*: *L.* (*V.*) *braziliensis*, *L.* (*V.*) *peruviana*, *L.* (*V.*) *guyanensis*, *L.* (*V.*) *panamensis*, and *L.* (*V.*) *lainsoni*. Reference strains for *L.* (*Leishmania*) *amazonensis*, *L.* (*L.*) *mexicana* and *L. infantum* (syn. *L. chagasi*) [20]–[21] were included as well. Species identification of all reference strains and isolates was previously performed by MLEE [3] and confirmed by MLST [18].

### Patients, Clinical Samples and Diagnosis

Clinical samples from 192 patients with leishmaniasis-like lesions were prospectively collected from the IMTAvH (n = 117) and the HMC (n = 75) in Lima, Peru. These clinics are the National Reference Centers for leishmaniasis diagnosis and treatment within the Peruvian Ministry of Health and Ministry of Defense, respectively. In total, lancet scrapings (n = 14) or punch biopsy samples (n = 178) were collected from both centers and processed for direct microscopic observation after Giemsa staining. For some patients, aspirate samples were collected from skin lesions using sterile technique and inoculated into Senekji's blood-agar medium as previously described [3]. In a subgroup of patients, the LST was performed as previously published [22]. Briefly, leishmanin antigen (0.1 ml) from the *L.* (*V.*) *guyanensis* strain LP52 (IPRN/PE/87/Lp52) was injected intradermally into the forearm, and the extent of induration and erythema measured after 48 h. The LST result was considered positive if the diameter of the induration was 5 millimeters or more. All specimens were processed for kDNA PCR, which is specific for the *Leishmania Viannia* complex [23], and the FRET-based real-time PCR assay. Overall, all *Leishmania* strains in this study (isolates and clinical samples) are considered geographically diverse since they were isolated from cases occurring across the Peruvian coast (Lima, Piura, La Libertad and Lambayeque), the highlands (Ancash, Apurimac, Cerro de Pasco, Cajamarca, Cuzco and Junín) and the Amazon rainforest (Amazonas, Huánuco, Loreto, Madre de Dios, San Martin and Ucayali). Furthermore, we also analyzed 13 *L.* (*V.*) *panamensis* strains (4 isolated from Ecuador and 9 from Colombia), but these results are not included in this manuscript.

### DNA Isolation

DNA was isolated from parasite culture or clinical samples using the QIAamp DNA mini kit (QIAGEN) following the manufacturer's instructions. DNA pellets were resuspended in Tris-EDTA buffer and quantified using a NanoDrop 1000 Spectrophotometer.

### kDNA PCR Conditions

Conventional PCRs for kDNA targeting mini-circles DNA were carried out to diagnose all specimens as a gold standard. This kDNA PCR can detect all *Leishmania* species from the *Viannia* complex [23] and has shown high sensitivity and specificity on different type of samples [24]. The PCRs were performed using a Gene Amp PCR System 9700 thermocycler (Applied Biosystems, Foster City, CA) in a total volume of 20 µl containing 4 µl DNA, 1X PCR buffer (Invitrogen), 0.5 µM of each primer (MP1-L: 5′-TAC TCC CCG ACA TGC CTC TG-3′ and MP3-H: 5′-GAA CGG GGT TTC TGT ATG C-3′), 1 U Taq DNA Polymerase (Invitrogen, Grand Island, NY), 1.5 mM MgCl_2_, and 125 µM of each dNTP. Initial denaturation at 94°C for 5 min was followed by 35 cycles of denaturation at 94°C for 45 sec, annealing at 58°C for 45 sec, and extension at 72°C for 60 sec; and a final extension at 72°C for 5 min. Amplified products were analyzed on 2% agarose gels; the expected product size is 70 bp.

### Real-time PCR Conditions

Conventional PCRs for *MPI* and *6PGD* genes were carried out prior to the nested real-time PCR amplifications in order to increase sensitivity of the assays. The initial PCRs were performed using a Gene Amp PCR System 9700 thermocycler (Applied Biosystems) in a total volume of 50 µl containing 5 µl DNA, 1X PCR buffer (Invitrogen), 1 µM of each primer (**Table 1**), 1.5 U Platinum *Taq* DNA Polymerase (Invitrogen), 1.5 mM MgCl_2_, and 200 µM of each dNTP. Initial denaturation at 94°C for 5 min was followed by 35 cycles of denaturation at 94°C for 45 sec, annealing at 57°C (for *MPI*) or 62°C (for *6PGD*) for 45 sec, and extension at 72°C for 90 sec; and a final extension at 72°C for 7 min for *MPI* or 5 min for *6PGD*. The subsequent PCR products were then used to perform the nested real-time PCR assays without further processing. Independent real-time reactions for *MPI* and *6PGD* genes were performed in a LightCycler 480 Instrument (Roche Applied Science). Reactions were carried out in a 20 µl total volume containing 1X LightCycler 480 Genotyping Master (Roche), 1.25 µM of forward primer, 0.25 µM of reverse primer, 0.18 µM of anchor probe, 0.18 µM of sensor probe (**Table 2**), and 5 µL of DNA (for reference strains used as positive controls) or PCR products. The amplification setting consisted of an initial denaturation at 95°C for 5 min followed by 45 cycles of denaturation at 95°C for 10 sec, annealing at 60°C for 20 sec (a single acquisition step) and extension at 72°C for 20 sec. After amplification, a melting curve analysis was performed by heating the real-time PCR products at 95°C for 10 sec, cooling at 50°C for 59 sec and then increasing the temperature to 80°C while continuously monitoring the fluorescence (one acquisition step per °C). Melting curves were analyzed using the LightCycler 480 Software Version 1.0 release 1.1.0.0520 (Roche Molecular System, Penzberg, Germany) to determine the species-specific melting temperatures (*Tm*). To calculate and enhance the visualization of the *Tm* values, melting peaks were derived from the initial melting curves (fluorescence [*F*] versus temperature [*T*] by plotting the negative derivative of fluorescence over temperature –d*F*/d*T* versus d*T*) [12]. A 483–670 nm filter combination was used for *MPI* while a 483–610 nm combination was used for *6PGD*. We followed all appropriate recommendations to avoid cross-contamination, including physical separation of PCR reaction and amplification products, use of UV light to eliminate DNA traces on work surface, aliquoting reagents and master mixes, etc [25]. For conventional PCR assays, *L.* (*V.*) *braziliensis* DNA (1 ng) was used as a positive control and nuclease-free water was used as a negative control. For real-time PCR assays, DNA (1 ng) from *L.* (*V.*) *braziliensis*, *L.* (*V.*) *peruviana*, *L.* (*V.*) *guyanensis*, *L.* (*V.*) *panamensis*, *L.* (*V.*) *lainsoni*, *L*. (*L.*) *amazonensis*, and *L.* (*L.*) *mexicana* strains were used as both references and positive controls and nuclease-free water was used as a negative control.

**Table 1:** Primers used in the conventional PCR assays for *MPI* and *6PGD* genes carried out prior to the nested real-time PCR assay.

Gene	Primer name	Sequence (5′ – 3′)	Source
*MPI*	MPI.ext.F	CCC TTT GGT TGT CGG T	[18]
	MPI.ext.R	TCA TAC GCA TAG GAG CA	[18]
*6PGD*	6PGD.909.F	CAA GGC GTT CCC TAC ATT C	This study
	6PGD.1537.R	TTG CGG TCG GGA CAA CTG G	This study

**Table 2:** Primers and labeled-probes used in the nested real-time PCR assays for *MPI* and *6PGD* genes.

Gene	Primer and probe name	Sequence (5′ – 3′)
*MPI*	MPI.1082.F	ACG CCC AAG TGG AAG GAT G
	MPI.1082.R	ACA CCA CTG TAC CGT TCA CC
	MPI.1082.anchor	TTC CAG ACA GAA GCC CAG CCC AAT CGT CGG – **fluorescein**
	MPI.1082.sensor	**red670** – GTC ACG GAG GTC GTC CCG CTT CCA G
*6PGD*	6PGD.1262.F	CAA GGA GAT GAA GGA GGG TC
	6PGD.1262.R	CTT GTC AAC ACG TTC GTA GC
	6PGD.1262.anchor	GCC AGG GAG GCA GTC ATC ACC G – **fluorescein**
	6PGD.1262.sensor	**red610** – AAC GAT ACA GCC GTG CTC GC

### MLST

Seventy-two clinical samples were assessed by MLST as described previously [8]. Briefly, *MPI* and *6PGD* specific primers were used to amplify each product individually. Direct sequencing was performed using the BigDye Terminator v3.1 cycle sequencing kit (Applied Biosystems) and analyzed on an ABI Prism 3100×l automated DNA sequencer (Applied Biosystems). Sequence analysis was performed using Sequencher v4.8 (Gene Codes Corporation, Ann Arbor, MI).

### Analytical Sensitivity Evaluation

PCR products of the *MPI* and *6PGD* genes of *L.* (*V.*) *braziliensis*, *L.* (*V.*) *peruviana*, *L.* (*V.*) *guyanensis*, and *L.* (*V.*) *panamensis* were purified and cloned into pGEM-T Easy Vector System (Promega). Serial dilutions from 10^6^ to 1 plasmid copy were evaluated in single and multiplexed reactions. The detection limit was defined as the minimum number of plasmids that could be amplified and correctly identified by melting curve analysis.

### Cost Analysis

Consumables, laboratory reagents and labor were considered in the cost estimations. Total costs per type of assay were calculated for a batch of 10 samples plus positive and negative controls and divided by 10 to estimate costs per individual sample. *MPI* and *6PGD* assays were considered as a combined analysis for cost analysis. Fixed costs such as facility space, electricity, air conditioning, etc., as well as costs and labor associated with DNA isolation were considered equivalent between the two methods and were not included for this analysis. All costs were obtained directly from U.S.-based manufacturers and shipping costs were not taken into consideration.

### Statistical Analysis

Data were analyzed using Stata v11.0 for Windows (Stata Corporation, College Station, TX). Descriptive statistics (mean, standard deviation, median, range) were calculated for continuous variables. Sensitivity, specificity and predictive values including 95% confidence intervals (95% CI) were estimated for all diagnostic tests using kDNA PCR as the gold standard. Statistical differences between these values were estimated using the exact McNemar test for matched observations. Categorical variables were assessed by proportions, and differences among the groups were compared using Fisher's exact chi-square analysis. *p*-values less than 0.05 were considered statistically significant.

## Results

### Development of the Real-Time PCR Assays

**Figure 1:**
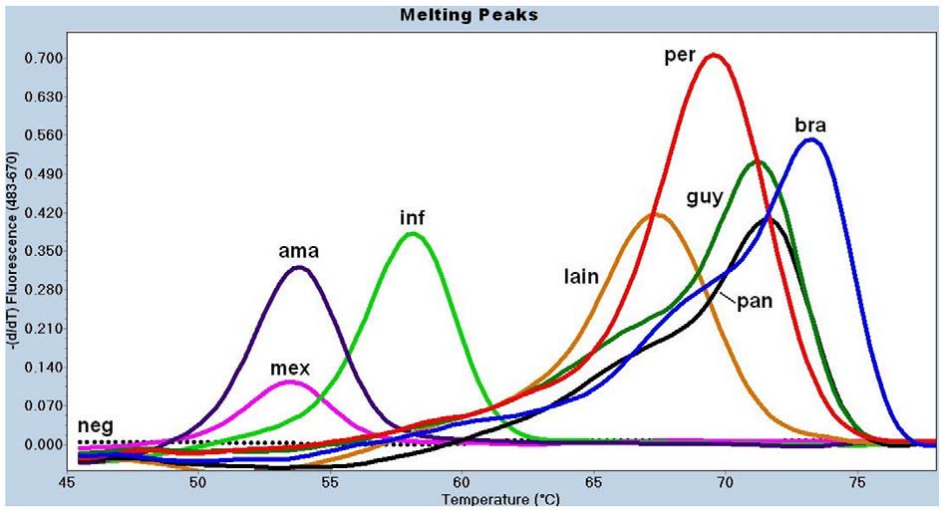
Melting curve analysis for the *MPI*-based real-time PCR assay on *Leishmania* reference strains. Five nanograms of DNA (references) or 5 ul of nested-PCR products (samples) were used as template to run the assay. The species designation is given based on the *Tm* calculated for each melting peak (point in the x axis for the peak of the curve). FRET probes on *L.* (*L.*) *amazonensis* (**ama**) or *L.* (*L.*) *mexicana* (**mex**) DNA are melted at lower temperature (53°C) while on *L.* (*V.*) *braziliensis* (**bra**) DNA are melted at the highest temperature (74°C). This assay could not differentiate between *L.* (*V.*) *guyanensis* (**guy**) and *L.* (*V.*) *panamensis* (**pan**) due to their overlapping *Tm* (72°C). All assessed *Leishmania* species yielded a melting curve. A straight dotted line corresponds to the negative controls (**neg**), DNA free or parasite-free human DNA. **inf** = *L. infantum*; **lain** = *L.* (*V.*) *lainsoni*; **per** = *L.* (*V.*) *peruviana*.

**Figure 2:**
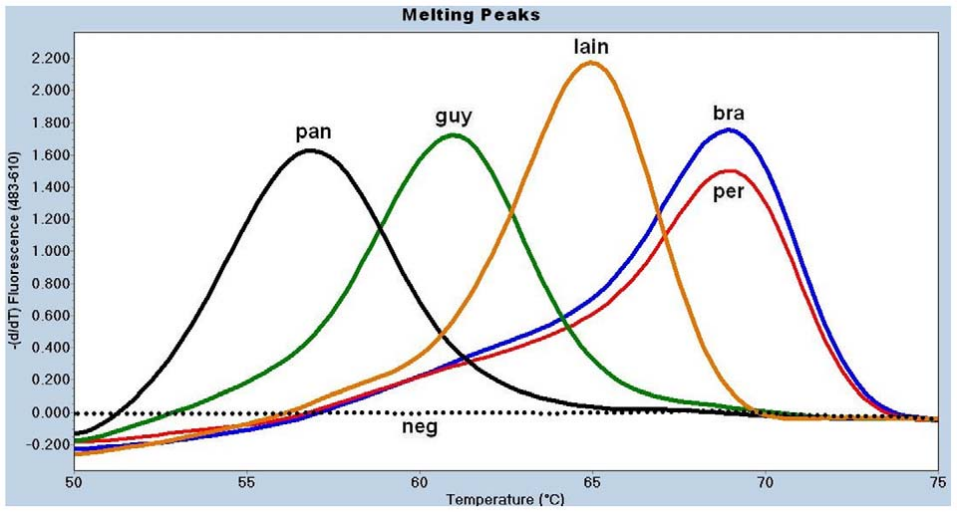
Melting curve analysis for the *6PGD*-based real-time PCR assay on *Leishmania* reference strains. Five nanograms of DNA (references) or 5 ul of nested-PCR products (samples) were used as template to run the assay. The species designation is given based on the *Tm* calculated for each melting peak (point in the x axis for the peak of the curve). FRET probes on *L.* (*V.*) *panamensis* (**pan**) DNA are melted at the lowest temperature (56°C) while on *L.* (*V.*) *braziliensis* (**bra**) or *L.* (*V.*) *peruviana* (**per**) DNA are melted at the highest temperature (68°C). Due to their overlapping *Tm*, this assay could not differentiate these two species. *L.* (*L.*) *amazonensis*, *L.* (*L.*) *mexicana* and *L. infantum* did not yield a melting curve. A straight dotted line corresponds to the negative controls (**neg**), DNA free or parasite-free human DNA. **guy** = *L.* (*V.*) *guyanensis*; **lain** = *L.* (*V.*) *lainsoni*.

We designed two sets of primers and hybridization probes capable of distinguishing previously identified single nucleotide polymorphisms (SNPs) in the *MPI* and *6PGD* loci [18]. Hybridization probes for the *MPI* gene were designed to detect the C1082G mutation, which differentiates between *L.* (*V.*) *braziliensis* and *L.* (*V.*) *peruviana* [19]. Several SNPs present in the region spanned by the anchor and sensor probes were found to differentiate *L.* (*V.*) *lainsoni* and *L. infantum* (syn *L. chagasi*). The probes for the *6PGD* gene were designed around the C1263G SNP, which differentiates between *L.* (*V.*) *guyanensis* and *L.* (*V.*) *panamensis* [18] (**Table 2**). Next, we performed melting curve analysis of the amplification products for both loci in order to assess whether intraspecific genetic variability affected correct species discrimination. For this purpose, we used five reference strains and 59 well-characterized *Leishmania* isolates that were previously differentiated by MLEE and MLST [3], [18]. The *MPI*-based assay yielded non-overlapping *Tm* values calculated for the melting peaks of *L.* (*V.*) *braziliensis* (74.0±0.1°C, mean ± standard deviation), *L.* (*V.*) *peruviana* (70.1±0.3°C), *L.* (*V.*) *lainsoni* (67.8±0.2°C), and *L. infantum* (58.7°C), allowing for a method of discrimination among these species (**Table 3** and **Figure 1**). However, this assay did not discriminate between *L.* (*V.*) *guyanensis* and *L.* (*V.*) *panamensis* or *L.* (*L.*) *amazonensis* and *L.* (*L.*) *mexicana* because their *Tm*s overlapped (**Table 3** and **Figure 1**). When the *6PGD*-based real-time PCR assay was used, we obtained clearly different *Tm*s for *L.* (*V.*) *guyanensis* (60.2±0.1°C), *L.* (*V.*) *panamensis* (56.2±0.7°C), and *L.* (*V.*) *lainsoni* (65±0.2°C), thus allowing identification of these species (**Table 3** and **Figure 2**). In addition to being able to differentiate five *Leishmania* species of the *Viannia* complex, the *MPI*-based real-time PCR gives distinct *Tm* for *L. infantum*, which belongs to the *Leishmania donovani* complex. However, this assay cannot distinguish between *L. (L.) amazonensis* and *L. (L.) mexicana*, two species of the *Leishmania mexicana* complex (**Figure 1**). Overall, the combined use of both *MPI*- and *6PGD*-based real-time PCR assays allows the correct identification of five New World *Leishmania* species of the *Viannia* complex and one species of *Leishmania donovani* complex (**Table 3** and **Figures 1** and **2**). Melting peaks were not observed for negative controls, nuclease-free water or non-*Leishmania* DNA, indicating the absence of primer-dimers formation (**Figures 1** and **2**). Thus, *Tm* values could not be calculated.

**Table 3:** Melting temperature (°C ± SD) calculated for the melting peaks detected in 65 *Leishmania* strain isolates.

Species	n	*MPI*	*6PGD*
*L.* (*V.*) *braziliensis*	19	74.0±0.1	68.2±0.2
*L.* (*V.*) *peruviana*	14	70.1±0.3	68.0±0.3
*L.* (*V.*) *guyanensis*	8	71.7±0.1	60.2±0.1
*L.* (*V.*) *panamensis*	10	71.6±0.1	56.2±0.7
*L.* (*V.*) *lainsoni*	6	67.8±0.2	65.0±0.2
*L. infantum*	1	58.7	ND*
*L.* (*L.*) *amazonensis*	6	53.5±0.1	ND
*L.* (*L.*) *mexicana*	1	53.6	ND

(*) ND = non detected.

### Analytical Sensitivity of the Real-Time PCR Assays

Following optimization of the real-time PCR conditions, we determined the analytical sensitivity of the assays. The *MPI*- and *6PGD*-based real-time PCR assays detected as few as one copy of plasmid when carried out in separate reaction tubes (data not shown). When the *MPI* and *6PGD* assays were multiplexed into a single reaction, the detection limit was 10–100 copies (data not shown).

### Cross-Reactivity of the Real-Time PCR Assays

No cross-reaction was observed when the two real-time PCR assays were used to test DNA from *Homo sapiens* (20 ng DNA), *Trypanosoma cruzi* (ATCC 30013 strain, American Type Culture Collection, 1–2 ng DNA), *Trypanosoma cruzi* Tulahuen strain (1–2 ng DNA), *Plasmodium falciparum* D6 strain (1–2 ng DNA), and *P. vivax* OBT9140 isolate (20 ng DNA).

### Sensitivity and Specificity with Clinical Specimens

A total of 192 specimens belonging to the same number of individuals with leishmaniasis-like lesions were evaluated (178 biopsy and 14 lesion scraping samples). Of these, 178 samples (92.7%) showed a positive result by at least one of the four diagnostic methods (microscopy, culture, LST and kDNA PCR). One hundred sixty-six samples (86.5%) were positive by kDNA PCR. When kDNA-PCR was used as the gold standard for leishmaniasis diagnosis, the real-time PCR had a sensitivity and specificity of 92% and 77%, respectively, and the positive and negative predictive values were 97% and 59%, respectively (**Table 4**). The sensitivity of the real-time PCR among kDNA PCR positive samples was superior to that of each of the other diagnostic tests, including microscopy (93% vs. 59%, *p*<0.001), culture (93% vs. 31%, *p*<0.001) and LST (93% vs. 68%, *p*<0.001) (**Table 4**). Stratification of the analysis by site revealed that the sensitivity of the real-time PCR tended to be higher among samples collected from IMTAvH compared to those collected at HMC (**Table 5**). The analysis of the discordant samples showed that fourteen samples were positive by kDNA PCR but negative by real-time PCR (false negative), and six samples were negative by kDNA PCR but positive by real-time PCR (false positive). Of the fourteen samples classified as false negatives, one was positive by microscopy and thirteen other samples were consistently negative by both microscopy and culture, likely indicative of low levels of parasites in these specimens (**Table S1**). The presence of PCR inhibitors was ruled out in these fourteen samples since DNA preparations in a 10-fold dilution were tested by PCR assays targeting two human housekeeping genes: *β-globin* and *Ribonuclease P*. While both undiluted and diluted samples were positive for *β-globin* and *Ribonuclease P*, all remained negative by the *MPI* and *6PGD* real-time PCR assays. Additionally, of the six specimens classified as false positive, four tested positive by microscopy or LST (**Table S2**), indicating that these four samples (two only detected by *6PGD* real-time PCR) likely were truly positive but not detected by kDNA PCR.

**Table 4:** Sensitivity, specificity and predictive values for all tested methods using kDNA PCR as the gold standard.

	kDNA PCR		% Sensitivity (95% CI)	% Specificity (95% CI)	% PPV (95% CI)	% NPV (95% CI)
	No. Positive	No. negative				
**Real-time PCR**						
Positive	152	6	92 (86–95)	77 (58–93)	96 (92–98)	59 (42–74)
Negative	14	20				
**Microscopy**						
Positive	89	5	59* (51–67)	78 (58–93)	95 (88–98)	23 (15–32)
Negative	62	18				
**Culture**						
Positive	36	2	31* (23–39)	89 (69–97)	95 (83–99)	17 (11–26)
Negative	82	17				
**LST**						
Positive	50	10	68* (57–77)	52 (32–72)	83 (72–91)	31 (19–48)
Negative	24	11				

(*) Statistically significant (*p*<0.001) when compared to real-time PCR by the exact McNemar's test. For this analysis, we considered only those samples that were processed by both assays being compared.

All samples were not assessed with all methods.

**Table 5:** Sensitivity and specificity of the real-time PCR assay compared to kDNA PCR stratified by site, type of sample and type of lesion.

Variable	Samples assessed	kDNA positives	n/N, % Sensitivity (95% CI)	n/N, % Specificity (95% CI)
**Site**				
IMTAvH	117	92	91/92, 99*(94–100)	19/25, 76(55–91)
HMC	75	74	61/74, 82(72–90)	1/1, 100(3–100)
**Type of sample**				
Biopsy	178	156	142/156, 91(85–95)	16/22, 73(50–89)
Scraping	14	10	10/10, 100(69–100)	4/4, 100(40–100)
**Form of lesion**				
Cutaneous	163	144	130/144, 90(84–95)	14/19, 74(49–91)
Mucocutaneous	29	22	22/22, 100(85–100)	6/7, 86(42–100)

(*) Statistically significant (*p*<0.001) by Chi-square test.

### *Leishmania* Species Identification

Among the 158 clinical samples that tested positive by real-time PCR, we were able to correctly identify 147 (93%) specimens to the species level. Four *Leishmania* species were identified among the IMTAvH samples: *L.* (*V.*) *peruviana* (43%), *L.* (*V.*) *braziliensis* (30%), *L.* (*V.*) *guyanensis* (12%), and *L.* (*V.*) *lainsoni* (3%). Two species were identified among the HMC samples: *L.* (*V.*) *braziliensis* (97%) and *L.* (*V.*) *guyanensis* (3%) (**Table 6**). In 11 IMTAvH clinical samples (11%), the *MPI* real-time PCR failed to amplify but *6PGD* real-time PCR did amplify. The *6PGD* real-time PCR results suggested infection by either *L.* (*V.*) *peruviana* or *L.* (*V.*) *braziliensis*. However, in the absence of *MPI* results, we could not discriminate between these *Leishmania* species (**Table 6**). In a subset of 72 clinical samples, regions of the *MPI* and *6PGD* genes were sequenced as previously described [18]. The concordance between the real-time PCR and the MLST analysis for species identification was 100% (**Table S3**). In summary, the real-time PCR assays reliably discriminated among New World *Leishmania* species that are highly prevalent in South America.

**Table 6:** *Leishmania* species identified by real-time PCR from individuals with suspected leishmaniasis from IMTAvH and HMC samples.

Species	IMTAvH	%	HMC	%
*L.* (*V.*) *braziliensis*	29	29.9	59	96.7
*L.* (*V.*) *peruviana*	42	43.3	–	–
*L.* (*V.*) *guyanensis*	12	12.4	2	3.3
*L.* (*V.*) *lainsoni*	3	3.1	–	–
BRA/PER*	11	11.3	–	–
Total	97	100	61	100

(*) No amplification for the *MPI* real-time PCR assay. *6PGD* real-time PCR results suggested infection by either *L.* (*V.*) *peruviana* or *L.* (*V.*) *braziliensis* but in the absence of MPI results, we could not discriminate between these two *Leishmania* species.

### Processing Time and Cost Analysis

We estimated the turnaround time and assay costs between the real-time PCR and the combination of kDNA PCR plus MLST for the specific identification of *Leishmania* species. The real-time PCR assay had lower costs and required less processing time compared to kDNA PCR plus MLST for the correct identification of *Leishmania* species (**Table 7**).

**Table 7:** Turnaround time and cost analysis per sample for the nested real-time PCR and MLST assays for the identification of *Leishmania* species from clinical specimens†.

Procedures\assays	kDNA-PCR/MLST (USD)	Nested real-time PCR (USD)
kDNA-PCR	1.3	NA^a^
PCR^b^	2.2	2.2
Bi-directional sequencing^b^	30.6	NA
real-time PCR^b^	NA	4.3
**Total cost (US dollars)**	**34.1**	**6.5**
**Turnaround time (hours)**	**20**	**6**

† Costs represent U.S. dollar amount per individual sample based on batch analysis of 10 samples plus one positive and one negative control.

a NA, not applicable.

b The cost includes the analysis of both *MPI* and *6PGD* genes per each processed sample.

## Discussion

In this report, we describe the development and evaluation of a real-time PCR assay for the diagnosis and characterization of NWTL species from tissue samples. This assay has several advantages: 1) It is highly sensitive, detecting as few as one copy of DNA, i.e. half of a diploid parasite genome, 2) It has higher sensitivity and specificity for the diagnosis of NWTLs when compared to conventional diagnostic tests, 3) It can reliably identify the infecting *Leishmania* species, including *L.* (*V.*) *braziliensis, L.* (*V.*) *panamensis L.* (*V.*) *peruviana, L.* (*V.*) *guyanensis*, and *L.* (*V.*) *lainsoni* (the most prevalent species causing NWTL in South America) [1]–[2], and 4) It is three times faster and five times less expensive compared to MLST for species identification. Because of these features, this real-time PCR assay can be a valuable contribution to the diagnosis and management of leishmaniasis in resource-limited settings.

In recent years, PCR has been established as the preferred method of *Leishmania* diagnosis and species identification due to its higher sensitivity and short turnaround time compared to traditional diagnostic tests [26]–[28]. We recently reported the presence of polymorphisms in various MLEE markers that could discriminate New World species of the *Viannia* complex [18]. Based on these mutations, we designed a real-time PCR assay to identify *Leishmania* species of the *Viannia* complex by virtue of their unique melting profiles. Melting curve analysis of the *MPI* and *6PGD* genes showed non-overlapping curves for five different *Leishmania* species among 59 isolates, suggesting that intraspecific genetic polymorphisms are not likely to affect correct species identification using this method. Since the strains belonged to patients from diverse regions of Peru (and a few from Ecuador and Colombia), we believe that intraspecific genetic variability will not affect correct species identification by the real-time PCR assay, thus allowing identification of geographically diverse strains of *Leishmania* species. This is further supported by sequencing analysis showing that the SNPs in the *MPI* and *6PGD* housekeeping genes are extremely well conserved even among genetically diverse strains [18].

The real-time PCR technique performed considerably better when compared to conventional diagnostic techniques such as microscopy, culture and LST in prospectively evaluated individuals with suspected leishmaniasis (n = 192). Additionally, our study confirmed the low sensitivity of microscopy, culture and LST, which ranged from 50 to 80% among various studies [29]–[31]. Given the high sensitivity values reported for kDNA PCR, we used this assay as a gold standard to assess the performance of the real-time PCR to diagnose NWTL [23], [29], [31]–[32], producing sensitivity and positive predictive values of 92% and 96%, respectively. Fourteen samples gave a positive result for kDNA PCR but were negative by the real-time PCR assay and were, therefore, classified as false negative. We ruled out the presence of PCR inhibitors in DNA preparations. The decreased sensitivity of the real-time PCR may be explained by larger amplicon size amplified for first-round PCR (∼1600 bp for the *MPI* locus versus ∼70 bp for the kDNA PCR), since partially-degraded DNA is more likely to amplify shorter amplicons compared to larger amplicons [33]. In keeping with this hypothesis, by shortening the first-round PCR of the MPI real-time PCR to 721 bp, we were able to detect 4 out of 10 initially-negative real-time PCR samples (data not shown). We are currently refining the assay using other strategies such as the use of alternative primers, shorter first-round PCR products and increasing the concentration of magnesium of the first PCR. The mini-circles of kDNA of *Leishmania* are also present as thousands of copies per parasite, whereas the *MPI* and *6PGD* genes are present as a single copy in the parasite's genome (*Leishmania braziliensis* GeneDB) [19], which could explain the higher sensitivity of the kDNA PCR assay over the nested PCR approach used in the real-time PCR assay [19], [34].

The specificity and negative predictive values of the real-time PCR were 77% and 59% respectively, when compared to kDNA PCR. However, 4 out of the 6 false positive samples had a positive result by either microscopy or LST. As a positive smear alone is not sufficient as criterion for a positive diagnosis of leishmaniasis, these results together with the available clinical diagnosis suggests that at least three of these six cases could be true positives that were missed by the kDNA PCR (**Table S2**). When these 3 samples were considered true positives, the adjusted specificity of the real-time PCR was 87%. These results highlight the importance of defining the right gold standard criteria for the diagnosis of leishmaniasis [24], [35] and support the hypothesis that the seemingly suboptimal specificity may be due to misclassification by the kDNA PCR rather than low specificity of the real-time PCR assay. The negative predictive value remained low (63%) after correcting for misclassification. However, this may be a reflection of the very high prevalence of leishmaniasis in the studied group (overall disease prevalence was ∼85%), composed mainly of suspected cases at reference centers. Further studies with larger sample size including subpopulations with lower prevalence of leishmaniasis are needed to confirm our findings and better estimate the sensitivity and specificity of the real-time PCR assay.

Several methods for diagnosis and species identification have been developed but most of these procedures only discriminate a few species within the *Viannia* complex [9], [13]–[17], [19]. While the real-time PCR assay we developed has proven to be highly sensitive for the diagnosis of NWTL, its main advantage resides in its ability to simultaneously identify up to five members of the *Viannia* subgenus. This assay reliably identified 64 archived parasite isolates and 147 prospectively collected skin samples at the species level with 100% concordance with MLST when compared in parallel in a subset of these samples. Besides the potential impact of this assay in the clinical setting, we successfully applied this real-time PCR assay for the identification and characterization of *Leishmania* species in field-collected sand fly specimens [36], underscoring the potential broad applicability of this assay.

One limitation of this study was the small number of negative samples included given the high prevalence of leishmaniasis in the study settings. This may have resulted in a less precise estimation of the predictive values since only few negative subjects were included in the analysis. An additional study is being planned to provide more accurate estimates of the specificity and the negative predictive value. A second limitation was that we were unable to apply all diagnostic tests simultaneously to all study participants. As a consequence, comparisons across diagnostic tests had to be done in subsets of subjects, which could limit the comparability of the tests. Finally, although we carried out this study at two major reference clinics for leishmaniasis, it remains possible that the strains included in this study do not represent all strains in Peru. Future studies in more diverse groups, including a larger pool of negative samples and belonging to wider geographic areas, are warranted to confirm the results of this study.

In summary, our real-time PCR assay can simultaneously diagnose New World leishmaniasis and identify the five causative *Leishmania* species most prevalent in South America, highlighting its potential regional applicability in all these countries. Thus, given its diagnostic performance, short turnaround time, scalability and relatively low costs, this assay could have great utility in the clinical setting and help to improve case management and direct appropriate therapy for patients with cutaneous and mucocutaneous leishmaniasis in resource-limited countries of South America.

## Supporting Information

Table S1 Results of conventional diagnostic tests for 14 clinical samples reported as false negatives. Conventional diagnostic tests and real-time PCR were negatives but the kDNA PCR assay was positive. Since melting peaks were not observed, *Leishmania* species could not be identified for these 14 samples.
(DOC)

Table S2 Results of conventional diagnostic tests for 6 clinical samples reported as false positives. At least one conventional diagnostic test and one real-time PCR assay were positives but the kDNA PCR assay was negative. Two samples yield melting peaks for both *MPI* and *6PDG* real-time PCR assays and the involved *Leishmania* species were identified. Four samples yield a melting peak for *6PGD* real-time PCR assay only and the involved *Leishmania* species were reported as BRA/PER.
(DOC)

Table S3 Seventy two clinical samples comparing *Leishmania* speciation by MLST and by our novel real-time PCR assay. A 100% concordance between MLST and real-time PCR assay was obtained when this subset of samples were compared in parallel. The shaded rows correspond to reference strains (sequenced in [18]) that were used as comparison. SNPs in *MPI* and *6PGD* that are detected by the real-time PCR probes are shown in bold. Dots indicate missing data. Dashes indicate that no sequencing was performed.
(DOC)
