# Supplementary material for: A FRET-Based Real-Time PCR Assay to Identify the Main Causal Agents of New World Tegumentary Leishmaniasis
Source: PLoS Negl Trop Dis. 2013 Jan 3;7(1):e1956. doi: 10.1371/journal.pntd.0001956 (PMC3536805; doi:10.1371/journal.pntd.0001956)
Supplement: Table S1 — Results of conventional diagnostic tests for 14 clinical samples reported as false negatives. Conventional diagnostic tests and real-time PCR were negatives but the kDNA PCR assay was positive. Since melting peaks were not observed, Leishmania species could not be identified for these 14 samples. (DOC) [file pntd.0001956.s001.doc]

**Table S1.** Results of conventional diagnostic tests for 14 clinical samples reported as false negatives. Conventional diagnostic tests and real-time PCR were negatives but the kDNA PCR assay was positive. Since melting peaks were not observed, *Leishmania* species could not be identified for these 14 samples.

|  |  |  |  |  |  |  |  |  |  |  |
| --- | --- | --- | --- | --- | --- | --- | --- | --- | --- | --- |
| **#** | **ID** | **Source** | **Type** | **Form** | **Culture** | **Smear** | **LST** | **kDNA** | **RT-PCR** | **Species** |
| 1 | LEH.1899 | HMC | biopsy | cutaneous | neg | NT | NT | pos | neg | unknown |
| 2 | LEH.1900 | HMC | biopsy | cutaneous | neg | neg | NT | pos | neg | unknown |
| 3 | LEH.1904 | HMC | biopsy | cutaneous | neg | pos | NT | pos | neg | unknown |
| 4 | LEH.1909 | HMC | biopsy | cutaneous | neg | neg | NT | pos | neg | unknown |
| 5 | LEH.1911 | HMC | biopsy | cutaneous | neg | neg | NT | pos | neg | unknown |
| 6 | LEH.1913 | HMC | biopsy | cutaneous | neg | neg | NT | pos | neg | unknown |
| 7 | LEH.1916 | HMC | biopsy | cutaneous | neg | NT | NT | pos | neg | unknown |
| 8 | LEH.1927 | HMC | biopsy | cutaneous | neg | neg | NT | pos | neg | unknown |
| 9 | LEH.1929 | HMC | biopsy | cutaneous | neg | NT | NT | pos | neg | unknown |
| 10 | LEH.1930 | HMC | biopsy | cutaneous | neg | neg | NT | pos | neg | unknown |
| 11 | LEH.1932 | HMC | biopsy | cutaneous | neg | neg | NT | pos | neg | unknown |
| 12 | LEH.1940 | HMC | biopsy | cutaneous | neg | neg | NT | pos | neg | unknown |
| 13 | LEH.1939 | HMC | biopsy | cutaneous | neg | NT | NT | pos | neg | unknown |
| 14 | LEH.2080 | IMTAvH | biopsy | cutaneous | neg | neg | neg | pos | neg | unknown |

pos = positive result, neg = negative result, NT= indicates that the specimen was not tested.
