# Supplementary material for: A FRET-Based Real-Time PCR Assay to Identify the Main Causal Agents of New World Tegumentary Leishmaniasis
Source: PLoS Negl Trop Dis. 2013 Jan 3;7(1):e1956. doi: 10.1371/journal.pntd.0001956 (PMC3536805; doi:10.1371/journal.pntd.0001956)
Supplement: Table S2 — Results of conventional diagnostic tests for 6 clinical samples reported as false positives. At least one conventional diagnostic test and one real-time PCR assay were positives but the kDNA PCR assay was negative. Two samples yield melting peaks for both MPI and 6PDG real-time PCR assays and the involved Leishmania species were identified. Four samples yield a melting peak for 6PGD real-time PCR assay only and the involved Leishmania species were reported as BRA/PER. (DOC) [file pntd.0001956.s002.doc]

**Table S2.** Results of conventional diagnostic tests for 6 clinical samples reported as false positives. At least one conventional diagnostic test and one real-time PCR assay were positive but the kDNA PCR assay was negative. Two samples yield melting peaks for both *MPI* and *6PDG* real-time PCR assays and the involved *Leishmania* species were identified. Four samples yield a melting peak for *6PGD* real-time PCR assay only and the involved *Leishmania* species were reported as BRA/PER.

| # | **ID** | **Type** | **Form** | **Culture** | **Smear** | **LST** | **kDNA** | **RT-PCR** | | **Dx** | **Peak** | **Species** |
| --- | --- | --- | --- | --- | --- | --- | --- | --- | --- | --- | --- | --- |
| **1** | **LEH.2063** | **biopsy** | **cutaneous** | **neg** | **neg** | **pos** | **neg** | | **pos** | **CL^1^** | **1.73** | **BRA / PER** |
| 2 | LEH.2064 | biopsy | mucocutaneous | NT | NT | neg | neg | | pos | CL | 0.71 | BRA / PER |
| **3** | **LEH.2066** | **biopsy** | **cutaneous** | **NT** | **pos** | **pos** | **neg** | | **pos** | **CL** | **1.15** | ***L. guyanensis*** |
| 4 | LEH.2067 | biopsy | cutaneous | neg | neg | neg | neg | | pos | CL | 0.55 | BRA / PER |
| **5** | **LEH.2070** | **biopsy** | **cutaneous** | **neg** | **pos** | **neg** | **neg** | | **pos** | **CL** | **1.59** | **BRA / PER** |
| **6** | **LEH.2089** | **biopsy** | **cutaneous** | **neg** | **pos** | **pos** | **neg** | | **pos** | **CL** | **1.73** | ***L. guyanensis*** |

pos = positive result, neg = negative result, NT = indicates that the specimen was not tested; Peak = peak fluorescence for real-time PCR (average peak height for positive controls = 1.7; average peak height for DNA-free negative controls = 0.1); BRA/PER = *L*.(*V*.) *braziliensis* or *L*.(*V*.) *peruviana* (only positive by 6PGD real-time PCR); Dx: final clinical diagnosis; CL: cutaneous leishmaniasis; ^1^This case showed positive response to antimonial therapy.
