# Supplementary material for: A FRET-Based Real-Time PCR Assay to Identify the Main Causal Agents of New World Tegumentary Leishmaniasis
Source: PLoS Negl Trop Dis. 2013 Jan 3;7(1):e1956. doi: 10.1371/journal.pntd.0001956 (PMC3536805; doi:10.1371/journal.pntd.0001956)
Supplement: Table S3 — Seventy two clinical samples comparing Leishmania speciation by MLST and by our novel real-time PCR assay. A 100% concordance between MLST and real-time PCR assay was obtained when this subset of samples were compared in parallel. The shaded rows correspond to reference strains (sequenced in [18]) that were used as comparison. SNPs in MPI and 6PGD that are detected by the real-time PCR probes are shown in bold. Dots indicate missing data. Dashes indicate that no sequencing was performed. (DOC) [file pntd.0001956.s003.doc]

**Table S3.** Seventy two clinical samples comparing *Leishmania* speciation by MLST and by our novel real-time PCR assay. A 100% concordance between MLST and real-time PCR assay was obtained when this subset of samples was compared in parallel. The shaded rows correspond to reference strains (sequenced in [18]) that were used for comparison. SNPs in *MPI* and *6PGD* that are detected by the real-time PCR probes are shown in bold. Dots indicate missing data. Dashes indicate that no sequencing was performed.

| **Code** |  |  | **MPI** |  |  | **MPI** |  |  | **6PGD** |  |  | **6PGD** | **MSLT** | **RT-PCR** |
| --- | --- | --- | --- | --- | --- | --- | --- | --- | --- | --- | --- | --- | --- | --- |
|  | **849** | **1005** | **1082** | **1098** | **1125** | **results** | **1243** | **1248** | **1262** | **1263** | **1416** | **results** |  |  |
| MHOM/BR/84/LTB300 | C | A | **C** | **G** | G | BRA | **G** | C | **G** | C | G | *BRA* | *L. braziliensis* | *L. braziliensis* |
| MHOM/PE/87/PAB2880 | C | A | **G** | G | G | *PER* | **G** | C | **G** | C | A | *PER* | *L. peruviana* | *L. peruviana* |
| MHOM/BR/75/M4147 | A | G | **C** | **T** | A | *GUY* | A | **T** | **A** | C | G | *GUY* | *L. guyanensis* | *L. guyanensis* |
| LEH2002 | C | A | G | G | G | *PER* | G | C | G | C | A | *PER* | *L. peruviana* | *L. peruviana* |
| LEH2015 | C | A | C | G | G | *BRA* | - | - | - | - | - | - | *L. braziliensis* | *L. braziliensis* |
| LEH2016 | C | A | G | G | G | *PER* | - | - | - | - | - | - | *L. peruviana* | *L. peruviana* |
| LEH2018 | C | A | G | G | G | *PER* | - | - | - | - | - | - | *L. peruviana* | *L. peruviana* |
| LEH2019 | C | A | G | G | G | *PER* | G | C | G | C | A | *PER* | *L. peruviana* | *L. peruviana* |
| LEH2028 | C | A | G | G | G | *PER* | - | - | - | - | - | - | *L. peruviana* | *L. peruviana* |
| LEH2029 | . | A | C | G | G | *BRA* | - | - | - | - | - | - | *L. braziliensis* | *L. braziliensis* |
| LEH2033 | C | A | C | G | G | *BRA* | - | - | - | - | - | - | *L. braziliensis* | *L. braziliensis* |
| LEH2034 | C | G | C | G | G | *BRA* | - | - | - | - | - | - | *L. braziliensis* | *L. braziliensis* |
| LEH2035 | C | A | G | G | G | *PER* | - | - | - | - | - | - | *L. peruviana* | *L. peruviana* |
| LEH2037 | C | A | C | G | G | *BRA* | - | - | - | - | - | - | *L. braziliensis* | *L. braziliensis* |
| LEH2038 | C | A | C | G | G | *BRA* | - | - | - | - | - | - | *L. braziliensis* | *L. braziliensis* |
| LEH2040 | C | A | C | G | G | *BRA* | - | - | - | - | - | - | *L. braziliensis* | *L. braziliensis* |
| LEH2044 | C | A | C | G | G | *BRA* | - | - | - | - | - | - | *L. braziliensis* | *L. braziliensis* |
| LEH2045 | C | A | C | G | G | *BRA* | - | - | - | - | - | - | *L. braziliensis* | *L. braziliensis* |
| LEH2046 | C | A | G | G | G | *PER* | - | - | - | - | - | - | *L. peruviana* | *L. peruviana* |
| LEH2047 | A | G | C | T | A | *GUY* | - | - | - | - | - | - | *L. guyanensis* | *L. guyanensis* |
| LEH2053 | C | A | G | G | G | *PER* | - | - | - | - | - | - | *L. peruviana* | *L. peruviana* |
| LEH2054 | C | A | G | G | G | *PER* | - | - | - | - | - | - | *L. peruviana* | *L. peruviana* |
| LEH2089 | - | - | - | - | - | - | A | T | A | C | G | *GUY* | *L.guyanensis* | *L.guyanensis* |
| LEH1865 | . | A | C | G | G | *BRA* | G | C | G | C | G | *BRA* | *L. braziliensis* | *L. braziliensis* |
| LEH1866 | C | A | C | G | G | *BRA* | G | C | G | C | G | *BRA* | *L. braziliensis* | *L. braziliensis* |
| LEH1867 | C | A | C | G | G | *BRA* | G | C | G | C | G | *BRA* | *L. braziliensis* | *L. braziliensis* |
| LEH1868 | C | A | C | G | G | *BRA* | G | C | G | C | G | *BRA* | *L. braziliensis* | *L. braziliensis* |
| LEH1869 | C | A | C | G | G | *BRA* | G | C | G | C | G | *BRA* | *L. braziliensis* | *L. braziliensis* |
| LEH1870 | C | A | C | G | G | *BRA* | G | C | G | C | G | *BRA* | *L. braziliensis* | *L. braziliensis* |
| LEH1871 | C | A | C | G | G | *BRA* | G | C | G | C | G | *BRA* | *L. braziliensis* | *L. braziliensis* |
| LEH1872 | C | A | C | G | G | *BRA* | G | C | G | C | G | *BRA* | *L. braziliensis* | *L. braziliensis* |
| LEH1873 | C | A | C | G | G | *BRA* | G | C | G | C | G | *BRA* | *L. braziliensis* | *L. braziliensis* |
| LEH1874 | C | A | C | G | G | *BRA* | G | C | G | C | G | *BRA* | *L. braziliensis* | *L. braziliensis* |
| LEH1875 | C | A | C | G | G | *BRA* | G | C | G | C | G | *BRA* | *L. braziliensis* | *L. braziliensis* |
| LEH1876 | C | A | C | G | G | *BRA* | G | C | G | C | G | *BRA* | *L. braziliensis* | *L. braziliensis* |
| LEH1877 | C | A | C | G | G | *BRA* | G | C | G | C | G | *BRA* | *L. braziliensis* | *L. braziliensis* |
| LEH1878 | C | A | C | G | G | *BRA* | G | C | G | C | G | *BRA* | *L. braziliensis* | *L. braziliensis* |
| LEH1879 | C | A | C | G | G | *BRA* | G | C | G | C | G | *BRA* | *L. braziliensis* | *L. braziliensis* |
| LEH1880 | . | A | C | G | G | *BRA* | G | C | G | C | G | *BRA* | *L. braziliensis* | *L. braziliensis* |
| LEH1881 | C | A | C | G | G | *BRA* | G | C | G | C | . | BRA/PER | *L. braziliensis* | *L. braziliensis* |
| LEH1882 | C | A | C | G | G | *BRA* | G | C | G | C | . | BRA/PER | *L. braziliensis* | *L. braziliensis* |
| LEH1883 | C | A | C | G | G | *BRA* | G | C | G | C | G | *BRA* | *L. braziliensis* | *L. braziliensis* |
| LEH1884 | . | A | C | G | G | *BRA* | G | C | G | C | G | *BRA* | *L. braziliensis* | *L. braziliensis* |
| LEH1885 | C | A | C | G | G | *BRA* | G | C | G | C | G | *BRA* | *L. braziliensis* | *L. braziliensis* |
| LEH1886 | C | A | C | G | G | *BRA* | G | C | G | C | G | *BRA* | *L. braziliensis* | *L. braziliensis* |
| LEH1887 | C | A | C | G | G | *BRA* | G | C | G | C | G | *BRA* | *L. braziliensis* | *L. braziliensis* |
| LEH1888 | . | A | C | G | G | *BRA* | G | C | G | C | . | BRA/PER | *L. braziliensis* | *L. braziliensis* |
| LEH1889 | C | A | C | G | G | *BRA* | G | C | G | C | G | *BRA* | *L. braziliensis* | *L. braziliensis* |
| LEH1890 | C | A | C | G | G | *BRA* | G | C | G | C | G | *BRA* | *L. braziliensis* | *L. braziliensis* |
| LEH1891 | C | A | C | G | G | *BRA* | G | C | G | C | G | *BRA* | *L. braziliensis* | *L. braziliensis* |
| LEH1892 | C | A | C | G | G | *BRA* | G | C | G | C | G | *BRA* | *L. braziliensis* | *L. braziliensis* |
| LEH1893 | C | A | C | G | G | *BRA* | G | C | G | C | G | *BRA* | *L. braziliensis* | *L. braziliensis* |
| LEH1894 | C | A | C | G | G | *BRA* | G | C | G | C | G | *BRA* | *L. braziliensis* | *L. braziliensis* |
| LEH1895 | . | A | C | G | G | *BRA* | G | C | G | C | G | *BRA* | *L. braziliensis* | *L. braziliensis* |
| LEH1896 | C | A | C | G | G | *BRA* | G | C | G | C | G | *BRA* | *L. braziliensis* | *L. braziliensis* |
| LEH1897 | C | A | C | G | G | *BRA* | G | C | G | C | G | *BRA* | *L. braziliensis* | *L. braziliensis* |
| LEH1898 | C | A | C | G | G | *BRA* | - | - | - | - | - | *-* | *L. braziliensis* | *L. braziliensis* |
| LEH1901 | . | A | C | G | G | *BRA* | G | C | G | C | . | BRA/PER | *L. braziliensis* | *L. braziliensis* |
| LEH1902 | . | A | C | G | G | *BRA* | - | - | - | - | - | *-* | *L. braziliensis* | *L. braziliensis* |
| LEH1903 | . | A | C | G | G | *BRA* | G | C | G | C | . | BRA/PER | *L. braziliensis* | *L. braziliensis* |
| LEH1905 | C | A | C | G | G | *BRA* | - | - | - | - | - | - | *L. braziliensis* | *L. braziliensis* |
| LEH1906 | C | A | C | G | G | *BRA* | - | - | - | - | - | - | *L. braziliensis* | *L. braziliensis* |
| LEH1907 | C | A | C | G | G | *BRA* | - | - | - | - | - | - | *L. braziliensis* | *L. braziliensis* |
| LEH1908 | C | A | C | G | G | *BRA* | - | - | - | - | - | - | *L. braziliensis* | *L. braziliensis* |
| LEH1910 | C | A | C | G | G | *BRA* | - | - | - | - | - | - | *L. braziliensis* | *L. braziliensis* |
| LEH1912 | C | A | C | G | G | *BRA* | - | - | - | - | - | - | *L. braziliensis* | *L. braziliensis* |
| LEH1914 | C | A | C | G | G | *BRA* | - | - | - | - | - | - | *L. braziliensis* | *L. braziliensis* |
| LEH1917 | C | A | C | G | G | *BRA* | - | - | - | - | - | - | *L. braziliensis* | *L. braziliensis* |
| LEH1918 | C | A | C | G | G | *BRA* | - | - | - | - | - | - | *L. braziliensis* | *L. braziliensis* |
| LEH1919 | C | A | C | G | G | *BRA* | - | - | - | - | - | - | *L. braziliensis* | *L. braziliensis* |
| LEH1920 | C | A | C | G | G | *BRA* | - | - | - | - | - | - | *L. braziliensis* | *L. braziliensis* |
| LEH1921 | C | A | C | G | G | *BRA* | - | - | - | - | - | - | *L. braziliensis* | *L. braziliensis* |
| LEH1922 | C | A | C | G | G | *BRA* | - | - | - | - | - | - | *L. braziliensis* | *L. braziliensis* |
| LEH1923 | A | G | C | T | A | *GUY* | - | - | - | - | - | - | *L. guyanensis* | *L. guyanensis* |
| LEH1925 | C | A | C | G | G | *BRA* | - | - | - | - | - | - | *L. braziliensis* | *L. braziliensis* |
